# Self-reported delays in care-seeking in West Africa during the first wave of the COVID-19 pandemic

**DOI:** 10.1186/s12913-023-09812-x

**Published:** 2023-07-22

**Authors:** Natalie E. Conboy, Andre Nickow, John Koku Awoonor-Williams, Lisa R. Hirschhorn

**Affiliations:** 1 Northwestern University Feinberg School of Medicine, IL Chicago, USA; 2 Northwestern University Global Poverty Research Lab, Evanston, IL USA; 3 Formerly of the Department of Policy, Planning, Monitoring and Evaluation, Ghana Health Service, Accra, Ghana; 4 Robert J. Havey Institute of Global Health, Chicago, IL USA

**Keywords:** Delays in care-seeking, COVID-19, Food insecurity, Ghana, Sierra Leone, Burkina Faso

## Abstract

**Background:**

The COVID-19 pandemic caused delays in care-seeking due to fears of infection and decreased healthcare access globally. These delays have been linked in some countries to COVID-19 perceptions, decreased income, and food insecurity, but little is known about patient-level factors for decreased care-seeking specifically at the beginning of COVID-19 in West Africa. Understanding these factors is important to identify those at highest risk and address healthcare-related barriers.

**Methods:**

This study used self-reported data from telephone surveys in a population-based sample in Burkina Faso (*n* = 1352), Ghana (*n* = 1621), and Sierra Leone (*n* = 1301) in May–June 2020. Questions assessed delays in care-seeking, sociodemographic variables, COVID-19 beliefs, and food insecurity. Bivariate analyses using chi-square and multivariate analyses using logistic regression were used to explore associations between factors and delays in care-seeking by country. Independent variables were chosen based on prior research suggesting that financial insecurity, older age, female sex, rural location, and COVID-related concerns are associated with delays.

**Results:**

Between March-June 2020, 9.9%, 10.6%, and 5.7% of participants in Burkina Faso, Ghana, and Sierra Leone, respectively, delayed care-seeking. Food insecurity was prevalent (21.8–46.1%) and in bivariate analyses was associated with delays in care-seeking in Burkina Faso and Ghana. Concern about risk of household contraction of COVID-19 was common (18.1–36.0%) and in Ghana and Sierra Leone was associated with delays in care-seeking in both bivariate and multivariate analyses. In bivariate analyses, females showed more delays in Burkina Faso, while age above 30 and urban location were associated with delays in Ghana. In multivariate analyses, food insecurity was associated with increased delayed care-seeking in Burkina Faso.

**Conclusions:**

Multiple factors were associated with delays in care-seeking early in the COVID-19 pandemic, with food insecurity and concerns about infection showing significant associations in multiple countries. These findings highlight the need to invest in clinic accessibility, community education, and financial assistance to address barriers in healthcare. While many delays have subsided since the initial phase of the COVID-19 pandemic, understanding factors associated with early disruptions of care-seeking at the patient and household level will inform strategies for maintaining healthcare access during future pandemics in West Africa.

**Supplementary Information:**

The online version contains supplementary material available at 10.1186/s12913-023-09812-x.

## Introduction

The COVID-19 pandemic resulted in delays in care-seeking behavior in countries globally [1, 2]. Particularly during the beginning of the pandemic in March 2020, there were significant decreases in primary and specialty care visits due to fears of COVID-19 infection [2–4], government restrictions on movement [1, 3, 5], and intentions of reducing the use of medical care to reserve capacity for the most severely ill [5]. Delays in care-seeking resulted in challenges to individual and public health, including reductions in primary care visits [6], immunizations [7–9], prenatal care [10–12], pediatric care [13], cancer screenings [5], mental health visits [5], and emergency care [14], all of which have long-reaching economic and social consequences.

Studies thus far have yielded mixed results regarding differences in care-seeking across countries and settings during the COVID-19 pandemic. Several studies have suggested that low-income countries were disproportionally affected by delays of care-seeking due to COVID-19, particularly early in the pandemic [15, 16]. Forty-five percent of low-income and 30% of low-middle income countries (compared to 23% globally) reported partial disruptions in at least three quarters of their health services [15]. While another large, multinational study found no clear relationship between healthcare disruptions and national wealth, it suggested that urban regions may have been more impacted [17].

Studies that have emerged during this pandemic have also shown mixed findings regarding what populations and risk factors have been linked to decreased care-seeking, particularly in communities that already experienced significant barriers to care-seeking prior to COVID-19. One study comparing pre- and post-COVID-19 care-seeking in slum communities in four countries saw major decreases in care-seeking related to healthcare cost increases, income decreases, limited transportation to healthcare, and high fears of COVID-19 infection [18]. Another community study in Portugal suggested that those with decreased income since COVID-19, with less confidence in the pandemic government response, and who identify as women were more likely to delay care-seeking [19]. In Ethiopia, there is evidence that those who experienced transportation difficulties, had strong fears of COVID-19, and were over the age of 60 years were much less likely to present to care [20]. Those who experienced food insecurity were significantly more likely to delay care-seeking in the United States during the first wave of COVID-19 [21].

However, many patient-level factors that may influence care-seeking remain unknown, specifically in West Africa, where there were several known risk factors for the underutilization of healthcare prior to COVID-19. Previous research in this region suggested that access to healthcare is generally hindered by lack of transportation [22], long distances from clinics [23–26], and location in rural areas [27]. Lack of health insurance [23, 28] and low income [29] are significant financial barriers, and other patient factors, such as older age [27] and being female [24, 30], have also been linked to lower use of healthcare. Like other areas, West Africa reported major decreases in healthcare utilization during COVID-19 [31], with demonstrated reasons for delayed care-seeking being distrust of medical facilities [32], fear of contracting COVID-19, and lack of face masks to enter buildings [33]. Recent research in West Africa has begun to elucidate specific risk factors for delays of care-seeking during COVID-19. In particular, evidence exists that women were more severely affected by lost employment and lockdown restrictions during the pandemic than men [34], likely affecting their ability to seek care. Pregnant women in Ghana also showed decreased likelihood of seeking hospital antepartum care, reporting COVID-19 fears about illness as well as concerns about food and income loss [35]. However, very little is known on how other preexisting risk factors or socioeconomic concerns (e.g. financial stability, food insecurity) affected care-seeking during the early COVID-19 pandemic in this region.

Evidence regarding access and delivery of care during this pandemic is still emerging. Globally, many countries developed increased resources for routine, non-COVID-19 care in response to the first wave of the pandemic, but there have been mixed results on the relationship between pandemic stage, patient access to care [36], and likelihood of seeking care [37, 38]. Some global studies showed utilization and perceived access to care in some countries improving or returning to near pre-pandemic levels after lifting nationwide lockdowns [39–42], while others showed lasting effects, particularly in acute care [43]. A multinational study including Ghana suggested regional variation in healthcare disruption patterns during the first year of the COVID-19 pandemic, with Ghanaian healthcare services largely returning to baseline at the end of 2020 except for emergency room and total outpatient visits, which remained lower than in the years prior [17]. Considering the ever-evolving impact of COVID-19, it remains important to understand the risk factors that most impacted early care-seeking, before healthcare systems and government programs were able to adapt to pandemic circumstances and aid those most at risk.

The Research for Effective COVID-19 Responses (RECOVR) survey was developed by Innovations for Poverty Action (IPA), a nonprofit organization, and administered via telephone in ten countries between May and June 2020 [44]. The purpose of the survey was to assess the early economic impact of COVID-19 in real time and identify potential policy implications across the world. The survey included data from Burkina Faso, Ghana, and Sierra Leone in West Africa, all of which reported their first COVID-19 cases during the second week of March 2020.

We analyzed the survey results to understand the rates of self-reported delays in care-seeking and explore factors linked to delays in Burkina Faso, Ghana, and Sierra Leone during the first wave of the COVID-19 pandemic. We used these results to describe variability in factors associated with delays in care-seeking in these West African countries that, while geographically close, differ in economic and human development, with Ghana having a substantially higher human development index. Our aim was to use early data from the COVID-19 pandemic to explore community-reported themes related to the challenges in care-seeking that emerged during this pivotal time. Understanding the impact of COVID-19 on care-seeking behavior in West Africa, particularly at the beginning of this pandemic, is ultimately important to inform early interventions to address barriers to care at the individual, household, and health system levels during current and future pandemics.

## Methods

### Country context: poverty, food insecurity, and COVID-19 response

Table 1 shows demographic and early COVID-19 data for the included countries [45–49]. Ghana is classified by the United Nations as having a medium human development index, while Burkina Faso and Sierra Leone are classified as having low human development indices [50].

**Table 1:** Development and public health statistics for Burkina Faso, Ghana, and Sierra Leone

	**Burkina Faso**	**Ghana**	**Sierra Leone**
**Population (millions)**	20.9	30.1	8.0
**Poverty rate (< $2.15/day)**	30.5%	25.3%	26.1%
**Rate of moderate to severe food insecurity**	52.6%	36.6%	86.7%
**Life expectancy at birth (years)**	62	64	55
**Reported COVID-19 cases through July 1**^**st**^**, 2020**	962	17,741	1462
**Mortality rate under 5 years of age (per 1000 live births)**	85	45	108

### Study population

The target population of the surveys was adults who were over 18 years of age, owned a mobile phone, and lived in Burkina Faso, Ghana, or Sierra Leone. Participants were contacted via random digit dialing of all possible mobile phone numbers based on national communications authority number allocation plans and stratified by mobile network operator market share in each country. Individuals with working numbers were invited to participate in the survey.

### Data collection

Details of the RECOVR survey can be found in Egger et al. [44]. Briefly, the surveys were conducted via telephone interview in June 2020 (Burkina Faso), May 2020 (Ghana), and May through June 2020 (Sierra Leone). This approach minimized person-to-person contact and allowed for a wide geographical sample during the evolving COVID-19 pandemic. Survey enumerators conducted the survey in the participant’s self-reported preferred language, and the survey generally took under 30 min to complete.

Surveys captured sociodemographic characteristics of the respondent and household, healthcare usage since the start of the pandemic, COVID-19 attitudes and prevention behavior, social safety net access measures, food insecurity, and financial health. Measures of COVID-19 attitudes and prevention strategies included questions asking how many days in the past week were spent at home, without leaving or receiving visitors; whether the respondent felt that they or any household members were at risk of contracting COVID-19; their perception of the government’s response to COVID-19; and their primary concern regarding the effects of COVID-19. Measures of food insecurity and financial health included changes in income since mid-March 2020 and days in the last week where they had to reduce number of meals, limit portion sizes, or avoid buying food due to lack of money.

Participants’ self-reported delay in care-seeking was measured by one question: “Have you or any other person in your household delayed, skipped or been unable to complete needed health care visits since the middle of March (i.e., since schools were closed by the government. We define our study’s primary outcome, which throughout the paper is referred to as a “delay in care-seeking,” as a positive response to this question, indicating a delay in deciding to seek care or an inability to access necessary healthcare visits, regardless of the reason for the delay or lack of access. If respondents reported a delay, they were asked, “For what reasons have you or any other members of your household delayed or skipped needed health care since the middle of March?” Participants were prompted for up to three reasons, which were coded to fit: cost (could not afford care; could not afford transportation); lack of available services (clinic was closed; clinic had a long wait time/was understaffed); concerns about COVID-19 infection; and other reasons (appointment forgotten; too busy; had to work; other). This subgroup that reported delays in care-seeking underwent a separate analysis.

### Analysis

All analyses were conducted in STATA 16.1. Participants with missing responses to the primary outcome of delay of care-seeking were excluded from analysis (see Figs. 1, 2, and 3 in Appendix A for consort diagrams of Burkina Faso, Ghana, and Sierra Leone, respectively).

Due to prior research finding associations between delays in care-seeking and rural environment or distance from care, we wished to include urban status as a variable to explore. Unlike Sierra Leone, the Burkina Faso and Ghana RECOVR surveys did not have a question explicitly assessing urban versus rural status. Because of this, participant location in these countries was manually dichotomized as urban and non-urban. We chose the two largest cities and their respective regions in each country and defined “urban” as reporting both living in one of those two regions and simultaneously reporting living in the largest city/town in their stated region. Urban was ultimately defined as reporting living in Hauts Bassins or Centre (Burkina Faso) or Greater Accra or Ashanti (Ghana) as well as living in the largest city/town in their region.

There were three food insecurity questions, which asked whether someone in their household had limited food portions, reduced the number of meals eaten, and/or been unable to buy food in the past seven days. We created a composite food insecurity variable and dichotomized food insecurity as having reported that someone in their household had limited portions, reduced the number of meals eaten, and/or been unable to buy food for at least one day in the past week. Change in income was assessed as earning “more, the same, or less pay" in the last week as compared to a typical week before mid-March 2020. Change in income was later manually dichotomized as earning as more/same income versus less/no income as compared to pre-March 2020.

The survey data set was weighted by participant gender and geographic unit; weighting methods are further described in Egger et al. [44]. Weighted data and percentages were used for all analyses; raw numbers and percentages of participants and unweighted analyses are listed in Appendix B. Initial bivariate chi-square analyses were conducted between delays in care-seeking and other variables of interest, including demographic information, COVID attitudes and prevention strategies, food insecurity, and changes in income. Variables with *p* values < 0.20 in bivariate analyses were included in multivariate logistical regressions for each country.

### Ethical approval

The initial RECOVR research was approved by IPA’s IRB Protocol 15608 (Burkina Faso), 15542 (Ghana), and 15592 (Sierra Leone). The Northwestern University IRB designated this study as non-human subjects research, as the publicly available dataset was deidentified. Those who participated in the survey gave informed consent and received phone credit worth approximately 90 U.S. cents for their participation.

## Results

### Demographics

Overall, participants aged 18–30 years represented 52.5–60.4% of the total sample across the three countries, and men represented 42.4–45.9% of the sample. Participants living in urban areas made up 60.5% of the sample in Burkina Faso, 22.9% in Ghana, and 83.7% in Sierra Leone (Table 2). Approximately 34.5–40.8% of respondents had less than a secondary education.

**Table 2:** Bivariate analyses, by country, of sociodemographic information, COVID-19 perceptions, food insecurity, and self-reported delays in care-seeking using chi-square tests

	***Burkina Faso***^a^			***Ghana***^a^			***Sierra Leone***^a^		
	**%**	**Delay (%)**	***p* value**	**%**	**Delay (%)**	***p* value**	**%**	**Delay (%)**	***p* value**
**Total**		9.9			10.6			5.7	
**Age**			0.16			0.04*			0.56
18–30	58.4	8.3		60.4	8.9		52.5	4.9	
31–40	24.4	13.1		24.2	14.2		31.1	7.2	
41 +	17.2	10.4		15.4	11.4		16.3	5.3	
**Male/Female**			0.002**			0.55			1.00
Male	42.4	6.7		45.9	10.0		45.5	5.7	
Female	57.6	12.2		54.1	11.0		54.5	5.7	
**Location**			0.94			0.04*			0.62
Urban	60.5	9.8		22.9	11.3		83.7	5.9	
Non-Urban	39.5	10.0		77.1	7.9		16.3	4.5	
**Highest level of education completed**			0.82			0.07			0.28
Less than secondary	34.5	9.2		35.3	10.5		40.8	5.9	
Secondary or vocational	27.0	10.9		35.6	8.3		34.4	4.2	
Post-secondary	38.4	9.7		29.1	13.4		22.5	8.1	
Other							2.3	0.0	
**Days in the past week staying at home all day, without going out at all and without receiving any visits**			0.81			0.29			0.64
Never (0)	35.0	8.8		21.4	9.2		28.4	5.5	
Once (1)	9.1	12.5		7.5	8.5		12.7	3.6	
Some days (2–3)	18.4	9.4		15.2	7.6		18.4	4.4	
Most days (4–6)	12.7	8.7		24.0	11.6		18.3	6.6	
Every day (7)	24.8	8.8		31.9	12.8		22.1	7.5	
**Feels that they or anyone in the household is at risk of contracting COVID-19**			0.15			0.004**			0.04*
No	64.0	8.9		74.0	8.8		81.9	4.9	
Yes	36.0	12.2		26.0	15.0		18.1	9.3	
**Perception of the government’s reaction to COVID-19 outbreak**			0.24			0.08			0.06
Much too extreme	7.2	12.8		3.2	11.6		3.2	16.1	
Somewhat too extreme	8.5	16.7		2.6	4.6		11.0	6.0	
Appropriate	64.6	8.5		69.3	10.4		77.0	5.5	
Somewhat insufficient	10.5	10.1		8.5	17.3		7.2	3.7	
Not sufficient	9.2	10.6		16.4	8.3		1.7	1.8	
**Amount of pay earned in the past week**^b^			0.43			0.55			0.13
More or the same income	63.7	10.2		27.9	11.7		18.3	3.4	
Less or no income	36.3	12.7		72.2	10.2		81.7	6.5	
**Days in the past week where they had to limit portion size at meal-times**		x̄ = 0.97 (*SE* = 0.07)	0.002**		x̄ = 2.20 (*SE* = 0.08)	0.07		x̄ = 2.38 (*SE* = 0.11)	0.04*
No	75.6	8.0		56.8	9.1		54.5	4.0	
Yes	24.5	15.6		43.2	12.5		45.5	7.7	
**Days in the past week where they had to reduce the number of meals eaten in a day**		x̄ = 0.94 (*SE* = 0.06)	0.002**		x̄ = 2.43 (*SE* = 0.09)	0.11		x̄ = 2.43 (*SE* = 0.12)	0.20
No	74.7	8.0		53.5	9.2		55.6	4.6	
Yes	25.3	15.4		46.5	12.1		44.5	7.0	
**Days in the past week where they were unable to buy food due to drop in household income**			0.003**			0.001**			0.28
No	52.4	6.9		41.0	7.3		28.9	4.3	
Yes	47.6	13.1		59.0	13.0		71.1	6.5	
**Food insecurity**^c^			0.0007**			0.007**			0.08
No	46.1	6.1		29.0	7.0		21.8	3.2	
Yes	54.0	13.1		71.1	12.0		78.2	6.4	

^*^p < 0.05

^**^p < 0.01

^a^Percentages adjusted by survey weights as previously described. For absolute numbers, see Appendix B

^b^Compared to a week in mid-March

^c^Smaller portions, reducing number of meals, or unable to buy food

### COVID attitudes, prevention strategies, and effects

In regard to COVID-19, 22.1–31.9% of respondents reported staying home all 7 days in the past week, with 65.0–78.6% staying home at least one day (Table 2). In terms of COVID-19 perceptions, 18.1–36.0% of respondents felt that they or their household members were at risk of contracting COVID-19, and 64.6–77.0% perceived the government’s reaction to the outbreak as appropriate. Self-reported financial issues related to the COVID-19 crisis were common, with 36.3% of participants in Burkina Faso, 72.2% in Ghana, and 81.7% in Sierra Leone reporting earning less or no income compared to a typical week before mid-March 2020. Food insecurity was also common, with 24.5–45.5% reported limiting food portions, 25.3–46.5% reporting reducing meals, and 47.6–71.1% being financially unable to buy food in the past week. Overall, 54.0% of respondents in Burkina Faso, 71.1% in Ghana, and 78.2% in Sierra Leone experienced at least one type of recent food insecurity.

### Delays in care-seeking

Table 2 presents descriptive statistics and primary bivariate analyses. Overall, 9.9% of participants in Burkina Faso, 10.6% in Ghana, and 5.7% in Sierra Leone reported delaying, skipping, or being unable to complete health care visits between mid-March 2020 and completing the survey (May and June 2020). Delays in care-seeking were more common in those who reported at least one form of food insecurity. In Burkina Faso, 13.1% of those unable to buy food delayed care-seeking, while 6.1% of those who did not report food insecurity delayed care-seeking (*p* < 0.001); in Ghana, 12.0% of those unable to buy food delayed care-seeking compared to 7.0% of those who did not report food insecurity (*p* < 0.01). Respondents who felt that they or their household members were at risk of contracting COVID-19 had significantly higher rates of delayed care-seeking in both Ghana and Sierra Leone (15.0% of those who felt at risk vs. 8.8% of those who did not in Ghana, *p* < 0.01; 9.3% vs. 4.9% in Sierra Leone, *p* < 0.05). In Ghana, respondents in urban locations reported more delays in care-seeking (11.3% of urban vs. 7.9% of non-urban, *p* < 0.05). Ghanaian respondents of older age also reported more delays (14.2% of 31- to 40-year-olds and 11.4% of those over 41 years old, as compared to 8.9% of those under 30 years old, *p* < 0.05). In Burkina Faso, being female was associated with increased delays (12.2% of females vs. 6.7% of males, *p* < 0.05), but this was not seen in Ghana or Sierra Leone. There were no significant associations between delays in care-seeking and level of education completed, days spent at home, perception of government reaction to COVID, or changes in income since mid-March 2020.

### Multivariate analysis

Factors associated with reported delays in care varied by country in the multivariate analyses (Table 3). In Burkina Faso, being female or being 31- to 40-years-old as compared to under 30-years-old was associated with higher rates of delay in care-seeking (OR = 1.84, 95% CI [1.09–3.12],* p* < 0.05; OR = 2.02, 95% CI [1.30–3.14]*, p* < 0.01). Additionally, participants who reported food insecurity had increased rates of delays in care-seeking only in Burkina Faso (OR = 2.14, 95% CI [1.29–3.55] *p* < 0.01). In Ghana, being 31- to 40-years-old was associated with increased delays in care-seeking compared to under 30-years-old (OR = 1.59, 95% CI [1.00–2.53]* p* < 0.05). In both Ghana and Sierra Leone, feeling at risk of contracting COVID was associated with delays in care-seeking (OR = 1.63, 95% CI [1.08–2.45], *p* < 0.05; OR = 2.17, 95% CI [1.06–4.47], *p* < 0.05, respectively).

**Table 3:** Multivariate logistic regression of associations of delays in care-seeking with sociodemographic information, COVID-19 beliefs, income, and food insecurity, by country

	**Burkina Faso**		**Ghana**		**Sierra Leone**	
**Variable**	**Adjusted OR [CI]**	***p* value**	**Adjusted OR [CI]**	***p* value**	**Adjusted OR [CI]**	***p* value**
**Age**					n/a^a^	
18–30	Reference	-	Reference	-	-	-
31–40	1.84 [1.09–3.12]	0.02*	1.59 [1.00–2.53]	0.048*	-	-
41 +	1.36 [0.69–2.66]	0.37	1.45 [0.84–2.53]	0.19	-	-
**Male/Female**			n/a		n/a	
Male	Reference	-	-	-	-	-
Female	2.02 [1.30–3.14]	0.002**	-	-	-	-
**Location**	n/a				n/a	
Non-Urban	-	-	Reference	-	-	-
Urban	-	-	0.70 [0.47–1.04]	0.08	-	-
**Highest level of education completed**	n/a				n/a	
Less than secondary	-	-	Reference	-	-	-
Secondary or vocational	-	-	0.89 [0.56–1.43]	0.63	-	-
Post-secondary	-	-	1.30 [0.79–2.12]	0.30	-	-
**Feels that someone household is at risk of contracting COVID-19**						
No	Reference	-	Reference	-	Reference	-
Yes	1.44 [0.89–2.34]	0.14	1.63 [1.08–2.45]	0.02*	2.17 [1.06–4.47]	0.03*
**Perception of government’s reaction to COVID-19 outbreak**	n/a					
Much too extreme	-	-	(Reference)	-	(Reference)	-
Somewhat too extreme	-	-	0.32 [0.06–1.75]	0.19	0.34 [0.09–1.30]	0.12
Appropriate	-	-	0.72 [0.28–1.81]	0.48	0.37 [0.11–1.17]	0.09
Somewhat insufficient	-	-	1.2 [0.42–3.39]	0.73	0.18 [0.03–0.93]	0.04*
Not sufficient	-	-	0.55 [0.20–1.52]	0.25	0.12 [0.01–1.30]	0.08
**Amount of pay earned in the past week**^b^	n/a		n/a			
More or the same income	-	-	-	-	Reference	-
Less or no income	-	-	-	-	0.56 [0.23–1.33]	0.19
**Food insecurity**^c^						
No	Reference	-	Reference	-	Reference	-
Yes	2.14 [1.29–3.55]	0.003**	1.46 [0.96–2.22]	0.08	1.47 [0.60–3.59]	0.39

^*^p < 0.05

^**^p < 0.01

^a^Not included due to lack of significance in bivariate analyses

^b^Compared to a week in mid-March

^c^Smaller portions, reducing number of meals, or unable to buy food

### Reasons for delays in care-seeking

Self-reported reasons for delaying care-seeking also varied by country (Table 4). In Burkina Faso, 11.5% of those who delayed care-seeking cited cost of the visit and/or transportation as a factor, and 85.3% cited concern over being infected with COVID-19; in Ghana, 27.8% cited cost, and 63.7% cited COVID-19 infection concern; while in Sierra Leone, 45.8% cited cost, and 56.6% cited concern. In both Ghana and Sierra Leone, experiencing food insecurity was linked to delaying care-seeking specifically due to cost. In Ghana, 35.3% of those experiencing food insecurity delayed care-seeking due to cost, compared to 16.7% of those who did not experience food insecurity (*p* < 0.05); and in Sierra Leone, 51.5% of those experiencing food insecurity delayed care-seeking due to cost, compared to 4.4% of those who did not experience food insecurity (*p* < 0.001).

**Table 4:** Bivariate analyses, by country, of factors associated with reasons for delays among those who delayed care-seeking using chi-square tests

	***Burkina Faso* (*n* = 114)**				***Ghana* (*n* = 168)**				***Sierra Leone* (*n* = 64)**			
	Cost (%)	*p* value	Concern (%)	*p* value	Cost (%)	*p* value	Concern (%)	*p* value	Cost (%)	*p* value	Concern (%)	*p* value
**Total**	11.5		85.3		27.8		63.7		45.8		56.6	
**Male/Female**		0.41		0.43		0.08		0.11		0.70		0.23
Male	7.7		81.2		35.4		56.0		42.5		46.0	
Female	13.1		86.9		21.9		69.7		48.6		65.6	
**Location**		0.98		0.35		0.97		0.89		0.43		0.36
Urban	11.4		88.2		27.6		64.8		43.2		59.7	
Non-Urban	11.7		81.0		27.8		63.5		63.5		36.5	
**Highest level of education completed**		0.15		0.34		0.15		0.39		0.32		0.56
Less than secondary	19.7		84.1		35.6		56.8		43.9		47.4	
Secondary or vocational	13.1		78.7		30.8		64.3		30.5		61.0	
Post-secondary	3.3		91.6		18.5		70.7		60.5		65.6	
**Food insecurity**^a^		0.11		0.99		0.02*		0.35		0.0001**		0.30
No	3.5		85.3		16.7		68.8		4.4		74.0	
Yes	14.7		85.3		35.3		60.3		51.5		54.3	

^*^p < 0.05

^**^p < 0.01

^a^Smaller portions, reducing number of meals, or unable to buy food

## Discussion

We found that there were significant population-level rates of self-reported delayed care-seeking during the first wave of the COVID-19 pandemic in these three countries in West Africa. In bivariate analyses, factors associated with increased reported delays in care-seeking in at least one country included being female, concern about someone in the household contracting COVID-19, and food insecurity. Multivariate analyses ultimately showed higher rates of delayed care-seeking by those with self-reported food insecurity in Burkina Faso as well as those with self-reported fears of household COVID-19 infection in both Ghana and Sierra Leone. Among the households who reported delays in care-seeking, both cost and fear of infection were broadly noted as reasons for delays in care-seeking by a large number of participants in all three countries.

Food insecurity was common among survey respondents and was frequently linked with delaying care-seeking and, among those delaying in Ghana and Sierra Leone, with financial reasons for the delays. There is newly emerging research investigating the relationship between food insecurity during COVID-19 and delayed care-seeking in other parts of the world. In the United States, those with food insecurity had significantly higher odds of delaying or forgoing medical care, including specifically due to cost concerns [21]. Additional studies have shown links between food insecurity and decreased antenatal care in Kenya [51] as well as delayed healthcare by parents of low-income families in California. Considering that a large majority of respondents in Ghana and Sierra Leone (72.2% and 81.7%, respectively) and a substantial minority in Burkina Faso (36.3%) reported income loss, it is possible that food insecurity served as an additional marker for economic vulnerability during the early lockdowns. Such a correlation between food insecurity and delays in care-seeking is particularly alarming considering the worsening of poverty and food insecurity since the beginning of the pandemic [52]; an estimated 25 million people in West Africa could not meet their food needs in 2021, a 34% increase from 2020 [53]. Early research during the COVID-19 pandemic suggested that poor and rural households were most at risk of significant worsening of food insecurity during the pandemic and that these effects have the potential to impact countries for up to 10 years [54]. Our data suggest that food insecurity may be linked to delays in care-seeking; thus, a major, lasting destabilization of food security related to COVID-19 presents a major threat to ensuring continued healthcare. Regarding the preservation of healthcare utilization during times of stress, our results support the need to address not just the direct medical impact of COVID-19 but also the economic and social impact of lockdowns and income loss, which ultimately may indirectly affect patients’ access to healthcare due to competing economic demands. Thus, coordinated interventions on a national and international scale that target the social determinants of health have the potential to reduce economic vulnerability to delays in care-seeking in future global health crises.

Participants’ perceived risk of themselves or their family members contracting COVID-19 was also significantly associated with decreased care-seeking behavior in Ghana and Sierra Leone. Our findings support existing evidence that fear of COVID-19 infection contributed to delays in care-seeking during the early COVID-19 pandemic in Ghana [55] and Sierra Leone [31]. Worries about COVID-19 infection and resulting decreases in medical care have been seen internationally [4, 56–60], with some studies suggesting fear of infection to be the prevailing reason for decreased care-seeking by patients [11]. Patients may not have felt safe visiting medical clinics or leaving the home; while decreased movement is beneficial in slowing spread of COVID-19, it has serious unintended medical and social consequences, such as reduced treatment of infectious diseases (e.g. tuberculosis) and chronic diseases like diabetes [61, 62]. Effective communication from healthcare workers [63] and public health authorities [38, 64] to the general public is essential to balancing the spread of COVID-19 with the necessary utilization of regular medical care, and information dissemination has and will continue to influence continued COVID-19 vaccine uptake. Ensuring adequate patient education and access to personal protective equipment is an important area of future study regarding patients’ perceived safety in medical settings.

Sierra Leone had a lower overall rate of delays in care than Burkina Faso and Ghana. Of note, Sierra Leone was one of the primary locations for the Ebola outbreak in 2014, resulting in the enhancement of disease surveillance and mitigation systems. It is possible that such developments helped mitigate the delay in healthcare-seeking due to effective communication and trust in healthcare authorities in Sierra Leone [65]. Sierra Leone had a higher reported percentage of urban participants (83.7% compared to 60.5% in Burkina Faso and 22.9% in Ghana), although this contrast may reflect the manual coding of non-urban versus urban in Burkina Faso and Ghana, which may have underestimated urban residence. Ultimately, we did not see a difference in reported delays in care by urban versus non-urban settings, contrasting some pre-COVID [24, 66] and post-COVID [16, 17] health services research that found that variance in healthcare usage based on distance from clinics or metropolitan area. Other factors may also have been important, including strength of the primary care system. For example, the Ghanaian healthcare system has increased care delivery through Community-based Health Planning and Services (CHPS) zones, which have allowed for mobilization and increased proximity of care [67], potentially decreasing need to delay care in the context of COVID-19. Further research should investigate variation in the impact of COVID-19 by rural versus urban status by specifically assessing location status.

In contrast to a number of telephone surveys that have included primarily men (e.g. 33% female [68]), our weighted population was majority female (54–58%), suggesting adequate sampling. Previous research suggests that females have higher rates of delay globally during pandemics [69], and more recent research in Switzerland, Kenya, and Ghana has also suggested a link between female gender and delays in healthcare during the current COVID-19 crisis [34, 35, 70, 71]. However, in our sample, only Burkina Faso showed an association between female gender and increased delays in care-seeking. While we did not explore further, a previous study in Burkina Faso suggested that women have lower bargaining power in their households and work environments, leading to less control over their healthcare usage [72], perhaps contributing to the association seen in our study. Studies directly comparing delays in care-seeking across countries will be able to better characterize these observed associations.

Bivariate analyses showed that in Ghana, older age groups showed increased rates of delays in care-seeking. We do not have the knowledge to understand the cause of these delays, but possible reasons include that during COVID-19, older people may have needed additional financial and social support, as they may have been disproportionately impacted by social distancing and the decreased wages of those in their social network [32, 73]. In contrast to studies pre-pandemic and some studies during the COVID-19 pandemic [16], we did not see any differences based on education. Previous studies have found that patients with lower education completion were more likely to present later to healthcare for a variety of health conditions [24, 66]. Recent studies of delays in patient presentation for medical conditions caused by COVID-19 have also found that those with lower education were more likely to delay care-seeking [70]. It is possible that in our study, food insecurity was a strong marker of economic vulnerability with a more robust link with delayed care as compared to education; however, there was still no link between delays in care-seeking and education level, even when controlling for other variables in multivariate analysis. Our sample had high proportions of each education level, but it is still possible that it was underpowered or that there is an unmeasured confounder masking a link between education and delayed care-seeking.

There were a number of limitations to our study. The survey data were limited to participants with a mobile phone, affecting generalizability of results for individuals with less access to technology and mobile phone coverage. However, 75% of Ghanaians aged 15 and older reported owning a cell phone for personal use [74]. Cell phone coverage is also high in Burkina Faso and Sierra Leone (106 and 86 mobile phones per 100 people, respectively) [75], although the survey sample of Sierra Leone was significantly more urban compared to the two other countries, making it less representative of the general population. A prior study of phone surveys in sub-Saharan Africa during COVID-19 found that while survey participants are typically older and more educated than the general population, weighting based on likelihood of being interviewed (using individual and household characteristics) improved gender and educational representativeness of the samples [76]. Our use of sampling weights thus is likely to have improved generalizability of our results.

Additionally, the surveys in Burkina Faso and Ghana did not explicitly assess urbanicity of participants. Our approach of inferring urban versus non-urban location based on reporting living in the largest city of certain regions may have led to misclassification by underestimating the number of people in urban environments but smaller cities. This notable limitation may have affected our power to detect a relationship between location and delays in care-seeking.

Importantly, the surveys were collected from participants at a single time point. The available data cannot determine causality and does not assess any relationship between delays in care-seeking and harmful outcomes such as missed preventative care, disease diagnoses, and treatment of illness. Additionally, all analyses of factors were done within countries without statistical comparison between countries. Further studies that assess socioecological factors and policy in greater detail are needed to statistically assess potential differences between countries.

Many countries recognized challenges related to food insecurity early during the COVID-19 pandemic and provided food and other support. Ongoing and future research should assess if and how these factors or rates in delayed care-seeking changed throughout the subsequent waves of the pandemic and as a result of changes in government supports, health system changes, and infection prevention responses. Our power to identify associated factors was low due to the low percentage of participants reporting a delay in care-seeking. This may have limited our ability to detect links between care-seeking and other factors, such as education. Finally, our ability to better understand the causes of delays in care-seeking was limited by the data available from survey questions, which did not delineate whether healthcare was needed in the studied time frame, and, if so, what were the types, length, and outcomes of delayed care-seeking. Further exploration of these causes as well as the purpose and consequences of the delayed care will be vital to recognize those most at risk for poor outcomes and inform to support uninterrupted care-seeking in future health system shocks.

In conclusion, we found that there were notable rates of self-reported delays in care-seeking and several factors that related to these delays during the early COVID-19 pandemic in West Africa. Food insecurity and perceived risk of developing COVID-19 infection had significant relationships with delays in care-seeking, with those reporting increased food insecurity and/or perceived risk of developing COVID-19 having higher rates of delays in care-seeking. Increasing trust and safety of the health systems and patient education while addressing social determinants of health, such as ability to access and afford care, is necessary to improve overall care-seeking before, during, and after future pandemics. Further investigation into these factors and the sustained public health impacts of COVID-19 is ongoing and will likely also show variations in healthcare access and delivery over time in the ever-evolving landscape of COVID-19.

## Supplementary Information

**Additional file 1:** **Appendix A.** Consort diagrams for data collection, by country. **Figure 1.** Consort diagram for Burkina Faso. **Figure 2.** Consort diagram for Ghana. **Figure 3.** Consort diagram for Sierra Leone.

**Additional file 2:** **Appendix B.** Unadjusted bivariate and multivariate analyses by country. **Table 4.** Unadjusted bivariate analyses, by country, of sociodemographic information, COVID-19 perceptions, food insecurity, and self-reported delays in care-seeking using chi-square tests. **Table 5.** Unadjusted multivariate logistic regression of associations of delays in care-seeking with sociodemographic information, COVID-19 beliefs, income, and food insecurity, by country. **Table 6.** Unadjusted bivariate country analyses of self-reported reasons for delays in care-seeking and access, sociodemographic information, and food insecurity among individuals who reported delays in care-seeking.

## Data Availability

The raw data analyzed during the current study are available in the Innovations for Poverty Action Dataverse (Harvard Dataverse) online repository (Burkina Faso: 10.7910/DVN/BGHJYK; Ghana: 10.7910/DVN/QWLV0M; Sierra Leone: 10.7910/DVN/KIPFWO). Further data can be requested from the corresponding author upon reasonable request.

## Abbreviations

CHPS: Community-based Health Planning and Services
IPA: Innovations for Poverty Action
RECOVR: Research for Effective COVID-19 Response
