# Supplementary material for: Self-reported delays in care-seeking in West Africa during the first wave of the COVID-19 pandemic
Source: BMC Health Serv Res. 2023 Jul 22;23:785. doi: 10.1186/s12913-023-09812-x (PMC10363320; doi:10.1186/s12913-023-09812-x)

Appendix A. Consort diagrams for data collection, by country.

Figure 1. Consort diagram for Burkina Faso.

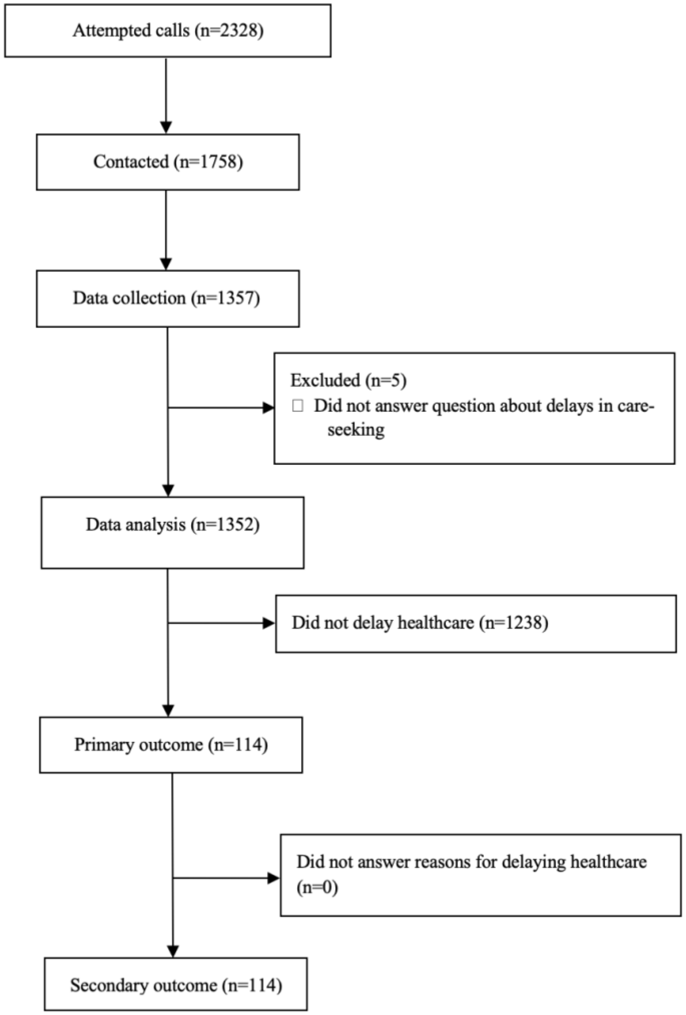

Figure 2. Consort diagram for Ghana

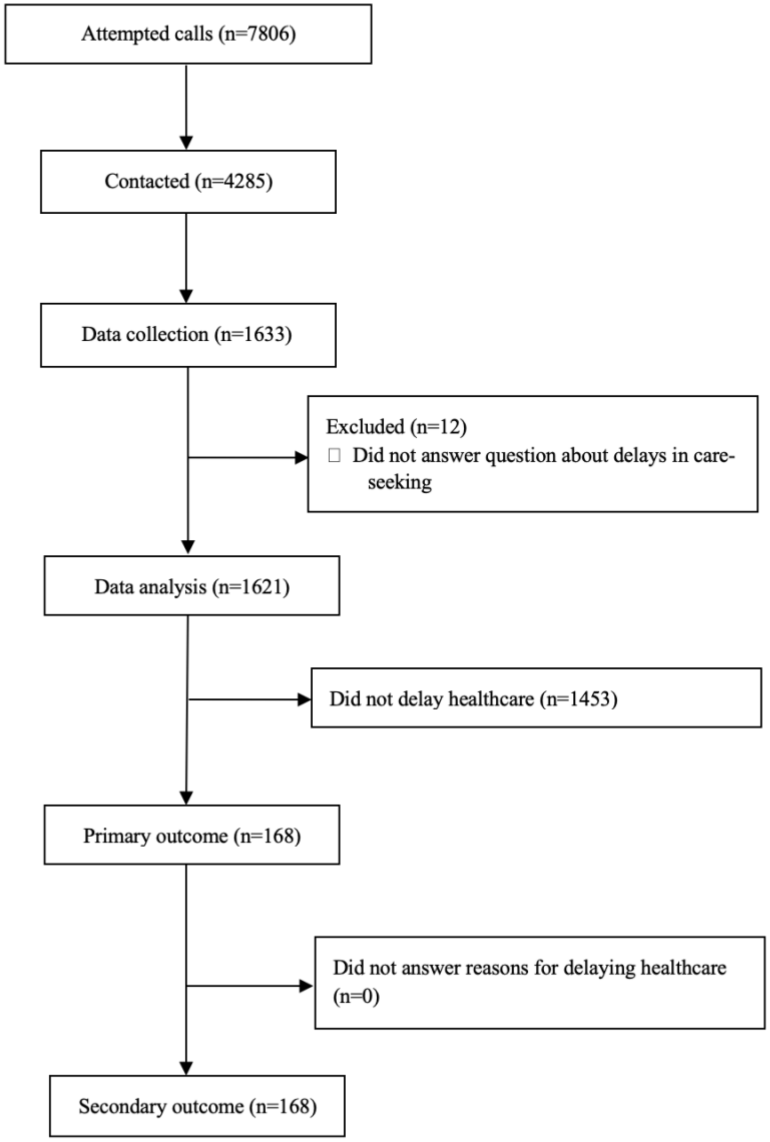

Figure 3. Consort diagram for Sierra Leone.

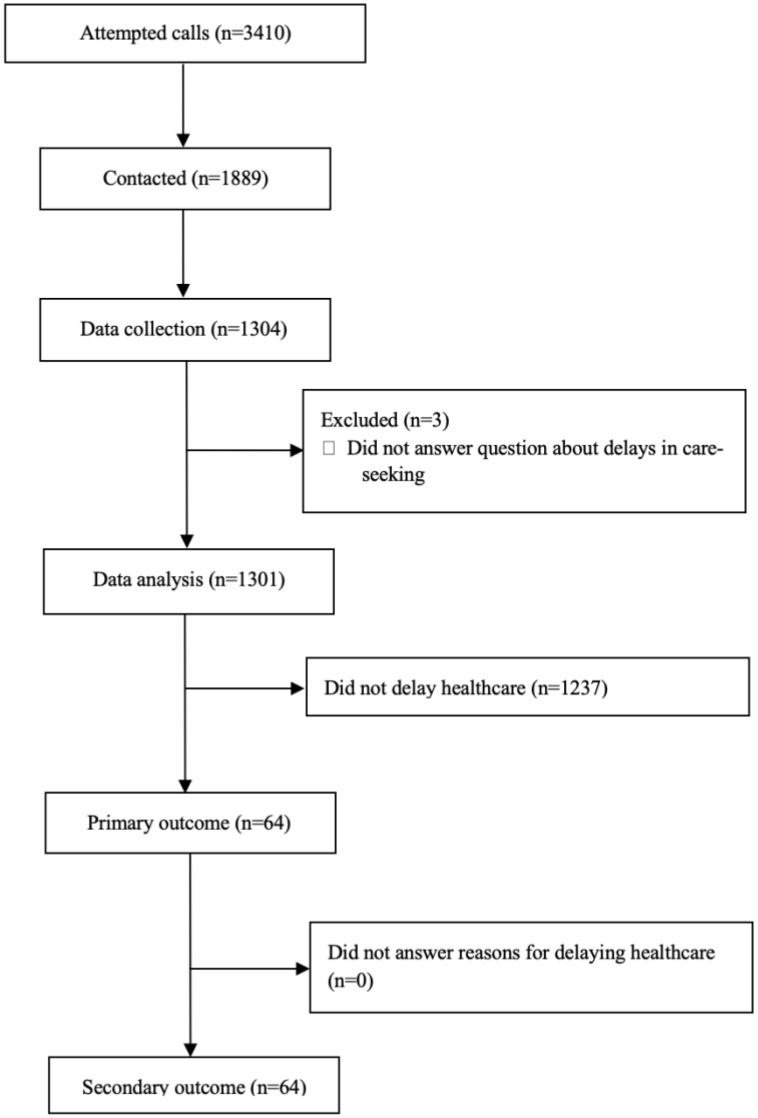

Supplement: Supplementary file 1 — Additional file 1: Appendix A. Consort diagrams for data collection, by country. Figure 1. Consort diagram for Burkina Faso. Figure 2. Consort diagram for Ghana. Figure 3. Consort diagram for Sierra Leone. [file 12913_2023_9812_MOESM1_ESM.pdf]
