# Supplementary material for: Self-reported delays in care-seeking in West Africa during the first wave of the COVID-19 pandemic
Source: BMC Health Serv Res. 2023 Jul 22;23:785. doi: 10.1186/s12913-023-09812-x (PMC10363320; doi:10.1186/s12913-023-09812-x)
Supplement: Supplementary file 2 — Additional file 2: Appendix B. Unadjusted bivariate and multivariate analyses by country. Table 4. Unadjusted bivariate analyses, by country, of sociodemographic information, COVID-19 perceptions, food insecurity, and self-reported delays in care-seeking using chi-square tests. Table 5. Unadjusted multivariate logistic regression of associations of delays in care-seeking with sociodemographic information, COVID-19 beliefs, income, and food insecurity, by country. Table 6. Unadjusted bivariate country analyses of self-reported reasons for delays in care-seeking and access, sociodemographic information, and food insecurity among individuals who reported delays in care-seeking. [file 12913_2023_9812_MOESM2_ESM.pdf]

Appendix B. Unadjusted bivariate and multivariate analyses by country

Table 4. Unadjusted bivariate analyses, by country, of sociodemographic information, COVID-19 perceptions, food insecurity, and self-reported delays in care-seeking using chi-square tests

	Burkina Faso				***Ghana***				***Sierra Leone***			
	**#**	**%**	**Delay**	***p* value**	**#**	**%**	**Delay**[1]	***p* value**	**#**	**%**	**Delay**	***p* value**
**Total**	114		8.43				10.4		64		4.9	
**Age**				**0.01****				0.13				0.54
18-30	749	55.4	6.8		914	56.4	9.2		648	49.7	4.3	
31-40	344	25.4	12.2		422	26.0	12.8		429	32.9	5.8	
41+	264	19.2	8.1		285	17.6	10.5		227	17.4	4.9	
**Gender**				**0.008****				0.27				0.69
Male	932	68.9	7.1		1000	61.7	9.7		844	64.9	5.1	
Female	420	31.1	11.4		621	38.3	11.4		457	35.1	4.6	
**Location**				0.84				0.32				0.62
Urban	901	66.4	8.3		1103	32.0	9.3		1176	90.4	5.0	
Non-Urban	456	33.6	8.7		518	68.0	10.9		125	9.6	4.0	
**Highest level of education completed**				0.94				0.49				0.07
Less than secondary	487	36.0	8.2		542	33.5	10.2		479	36.9	4.8	
Secondary or vocational	338	25.0	8.9		584	36.1	9.4		484	37.2	3.9	
Post-secondary	527	39.0	8.4		491	30.4	11.6		297	22.9	7.4	
Other									40	3.1	0.0	
**Days in the past week staying at home all day, without going out at all and without**				0.51				0.20				0.91

**receiving any visits**												
Never (0)	515	38.1	7.0		366	22.7	9.6		401	30.8	5.5	
Once (1)	139	10.3	9.4		137	8.5	7.3		177	13.6	5.1	
Some days (2-3)	231	17.1	7.8		266	16.5	8.7		235	18.1	4.7	
Most days (4-6)	159	11.8	10.1		360	22.3	10.3		250	19.2	5.2	
Every day (7)	308	22.8	10.6		486	30.1	13.0		238	18.3	3.8	
**Feels that they or anyone in the household is at risk of contracting COVID-19**				0.13				**0.004****				**0.000****
No	777	60.9	7.7		1158	73.5	8.9		1039	80.2	3.9	
Yes	500	39.2	10.2		418	26.5	13.9		257	19.8	9.3	
**Perception of the government's reaction to COVID-19 outbreak**				0.10				0.35				**0.01****
Much too extreme	98	7.3	13.3		53	3.4	13.2		48	3.7	14.6	
Somewhat too extreme	109	8.1	11.9		40	2.6	5.0		164	12.7	7.9	
Appropriate	855	63.2	7.1		1078	68.7	10.1		944	73.1	4.1	
Somewhat insufficient	145	10.7	7.6		145	9.2	14.5		109	8.4	3.7	
Not sufficient	145	10.7	11.0		254	16.2	9.8		27	2.1	3.7	
**Amount of pay earned in the past week**[2]				0.29				0.77				0.38
More or the same income	462	61.0	10.8		297	28.1	9.8		201	19.8	4.0	

Less or no income	295	39.0	8.5		761	71.9	10.4		813	80.2	5.5	
**Days in the past week where they had to limit portion size at meal-times**				**0.000****				**0.001****				**0.02***
No	1009	74.4	6.7		925	57.1	8.2		733	56.2	3.7	
Yes	348	25.6	13.4		696	42.9	13.2		571	43.8	6.5	
**Days in the past week where they had to reduce the number of meals eaten in a day**				**0.002****				**0.01****				**0.02***
No	1006	74.1	7.1		860	53.1	8.6		747	57.3	3.8	
Yes	351	25.9	12.4		761	47.0	12.4		557	42.7	6.5	
**Days in the past week where they were unable to buy food due to drop in household income**				**0.004****				**0.001****				0.21
No	691	52.4	6.4		674	42.9	7.6		385	30.4	3.9	
Yes	629	47.7	10.8		898	57.1	12.7		880	69.6	5.6	
**Food insecurity[3]**				**0.000****				**0.009****				0.10
No	624	46.0	5.5		502	30.7	7.4		315	24.2	3.2	
Yes	733	54.0	11.0		1131	69.3	11.7		989	75.8	5.5	

*$p < 0.05$

** $p < 0.01$

[2]Compared to a week in mid-March

[3]Smaller portions, reducing number of meals, or unable to buy food

Table 5. Unadjusted multivariate logistic regression of associations of delays in care-seeking with sociodemographic information, COVID-19 beliefs, income, and food insecurity, by country

	Burkina Faso		Ghana		Sierra Leone	
**Variable**	**Adjusted OR [CI]**	***p* value**	**Adjusted OR [CI]**	***p* value**	**Adjusted OR [CI]**	***p* value**
**Age**					n/a[1]	
18-30 (Reference)	-	-	-	-	-	-
31-40	2.06 [1.32-3.21]	**0.001****	1.47 [0.99-2.17]	**0.05***	-	-
41+	1.29 [0.74-2.23]	0.37	1.32 [0.83-2.09]	0.24	-	-
**Gender**			n/a		n/a	
Male (Reference)	-	-	-	-	-	-
Female	1.76 [1.17-2.65]	**0.01****	-	-	-	-
**Location**	n/a				n/a	
Non-Urban (Reference)	-	-	-	-	-	-
Urban	-	-	0.89 [0.62-1.28]	0.53	-	-
**Highest level of education completed**	n/a				n/a	
Less than secondary (Reference)	-	-	-	-	-	-
Secondary or vocational	-	-	1.08 [0.71-1.65]	0.73	-	-
Post-secondary	-	-	1.15 [0.74-1.78]	0.53	-	-
**Feels that someone household is at risk of**						

**contracting COVID-19**						
No (Reference)	-	-	-	-	-	-
Yes	1.76 [1.17-2.65]	**0.01****	1.56 [1.09-2.23]]	**0.02***	2.21 [1.22-4.01]	**0.01****
**Perception of government's reaction to COVID-19 outbreak**	n/a					
Much too extreme (Reference)	-	-	-	-	-	-
Somewhat too extreme	-	-	0.31 [0.06-1.60]	0.16	0.50 [0.17-1.46]	0.21
Appropriate	-	-	0.61 [0.27-1.41]	0.25	0.28 [0.11-0.72]	**0.01****
Somewhat insufficient	-	-	0.90 [0.35-2.31]	0.83	0.18 [0.04-0.79]]	**0.02***
Not sufficient	-	-	0.61 [0.24-1.52]	0.29	0.27 [0.03-2.46]	0.25
**Amount of pay earned in the past week[2]**	n/a		n/a			
More or the same income (Reference)	-	-	-	-	-	-
Less or no income	-	-	-	-	0.81 [0.36-1.78]	0.60
**Food insecurity[3]**						
No (Reference)	-	-	-	-	-	-

Yes	2.04 [1.34-3.13]	**0.001****	1.56 [1.10-2.21]	**0.01****	1.61 [0.73-3.52]	0.24

[1]Not analyzed due to lack of significance in bivariate analyses
[2]Compared to a week in mid-March
[3]Smaller portions, reducing number of meals, or unable to buy food
*$p \leq 0.05$
** $p \leq 0.01$

Table 6. Unadjusted bivariate country analyses of self-reported reasons for delays in care-seeking and access, sociodemographic information, and food insecurity among individuals who reported delays in care-seeking

	Burkina Faso (n=114)					Ghana (n=168)					Sierra Leone (n=64)				
	#	Cost	*p* value	Concern	*p* value	#	Cost	*p* value	Concern	*p* value	#	Cost	*p* value	Concern	*p* value
**Total**		11.5		85.3		49	29.2		63.1			40.6		50.0	
**Gender**			0.41		0.37			**0.03***		0.11			0.41		0.06
Male	66	6.1		78.8		97	35.4		56.0		43	44.2		41.9	
Female	48	14.6		85.4		71	21.9		69.7		21	33.3		66.7	
**Location**			0.68		0.15			0.97		0.89			0.98		0.64
Urban	75	8.0		85.3		120	27.6		64.8		59	40.7		49.2	
Non-Urban	39	12.8		74.4		48	27.8		63.5		5	40.0		60.0	
**Highest level of education completed**			0.10		0.40			0.15		0.39			0.98		0.56
Less than secondary	40	15.0		84.1		55	35.6		56.8		23	39.1		43.5	
Secondary or vocational	30	13.3		73.3		55	30.8		64.3		13	42.1		47.4	
Post-secondary	44	2.3		85.0		57	18.5		70.7		28	40.9		59.1	
**Food insecurity[1]**			0.11		0.89			**0.02***		0.35			**0.03***		0.17
No	34	2.9		82.4		53	16.7		68.8		10	10.0		70.0	
Yes	80	12.5		81.3		115	35.3		60.3		54	46.3		46.3	

*$p < 0.05$
** $p < 0.01$
[1]Smaller portions, reducing number of meals, or unable to buy food
